# Novel deletion alleles carrying *CYP21A1P*/*A2 *chimeric genes in Brazilian patients with 21-hydroxylase deficiency

**DOI:** 10.1186/1471-2350-11-104

**Published:** 2010-06-29

**Authors:** Fernanda B Coeli, Fernanda C Soardi, Renan D Bernardi, Marcela de Araújo, Luciana C Paulino, Ivy F Lau, Reginaldo J Petroli, Sofia HV de Lemos-Marini, Maria TM Baptista, Gil Guerra-Júnior, Maricilda P de-Mello

**Affiliations:** 1 Laboratório de Genética Molecular Humana, Centro de Biologia Molecular e Engenharia Genética, Universidade Estadual de Campinas, Campinas, SP, Brasil; 2 Departamento de Pediatria/Centro de Investigação em Pediatria, Faculdade de Ciências Médicas, Universidade Estadual de Campinas, Campinas, SP, Brasil; 3 Departamento de Clínica Médica, Disciplina de Endocrinologia, Faculdade de Ciências Médicas, Universidade Estadual de Campinas, Campinas, SP, Brasil

## Abstract

**Background:**

Congenital adrenal hyperplasia due to 21-hydroxylase deficiency is caused by deletions, large gene conversions or mutations in *CYP21A2 *gene. The human gene is located at 6p21.3 within a *locus *containing the genes for putative serine/threonine Kinase *RP*, complement *C4*, steroid 21-hydroxylase *CYP21 *tenascin *TNX*, normally, in a duplicated cluster known as RCCX module. The *CYP21 *extra copy is a pseudogene (*CYP21A1P*). In Brazil, 30-kb deletion forming monomodular alleles that carry chimeric *CYP21A1P/A2 *genes corresponds to ~9% of disease-causing alleles. Such alleles are considered to result from unequal crossovers within the bimodular *C4/CYP21 locus*. Depending on the localization of recombination breakpoint, different alleles can be generated conferring the locus high degree of allelic variability. The purpose of the study was to investigate the variability of deleted alleles in patients with 21-hydroxylase deficiency.

**Methods:**

We used different techniques to investigate the variability of 30-kb deletion alleles in patients with 21-hydroxylase deficiency. Alleles were first selected after Southern blotting. The composition of *CYP21A1P/A2 *chimeric genes was investigated by ASO-PCR and MLPA analyses followed by sequencing to refine the location of recombination breakpoints. Twenty patients carrying at least one allele with *C4/CYP21 *30-kb deletion were included in the study.

**Results:**

An allele carrying a *CYP21A1P/A2 *chimeric gene was found unusually associated to a *C4B/C4A Taq *I 6.4-kb fragment, generally associated to *C4B *and *CYP21A1P *deletions. A novel haplotype bearing both p.P34L and p.H62L, novel and rare mutations, respectively, was identified in exon 1, however p.P30L, the most frequent pseudogene-derived mutation in this exon, was absent. Four unrelated patients showed this haplotype. Absence of p.P34L in *CYP21A1P *of normal controls indicated that it is not derived from pseudogene. In addition, the combination of different approaches revealed nine haplotypes for deleted 21-hydroxylase deficiency alleles.

**Conclusions:**

This study demonstrated high allelic variability for 30-kb deletion in patients with 21-hydroxylase deficiency indicating that a founder effect might be improbable for most monomodular alleles carrying *CYP21A1P/A2 *chimeric genes in Brazil.

## Background

Congenital adrenal hyperplasia (CAH) is an autosomal recessive inborn error of metabolism due to the deficiency of one of the enzymes involved in the adrenal steroidogenesis [1]. More than 95% of the cases are due to deficiency of 21-hydroxylase enzyme (21-OHD; OMIM +201910). Diminished or absent 21-hydroxylase activity leads to reduction or abolishment of cortisol and aldosterone syntheses, consequently, an over production of androgens occurs [1,2]. Different clinical presentations are classified in classical form that can be salt-wasting (SW) or simple-virilizing (SV), and late-onset non-classical (NC) form [1-3]. Mutations on *CYP21A2 *gene, which encodes the enzyme, are responsible for the disease [2,3].

The *CYP21A2 *gene and *CYP21A1P*, its pseudogene, are located within the class III locus of the human major histocompatibility complex at the short arm of chromosome 6 [4,5]. Both have 10 exons, except that *CYP21A1P *is inactive due to deleterious mutations [6,7]. Approximately 30 kb separate *CYP21A2 *from *CYP21A1P*. The genes of the fourth component of complement *C4A *and *C4B*, also map to this locus and are located, respectively, upstream and downstream to the *CYP21A1P *[4,5].

In addition other genes, such as *RP1 *and *TNXB *and their truncated gene fragments or pseudogenes, *RP2 *and *TNXA*, are present within this genetic unit [8]. Each *RP*, *C4*, *CYP21*, *TNX *copy together forms the RCCX module [8] that maps within approximately 30 kb [9-11]. In general, 70% of alleles are bimodular (two RCCX units) in most populations [12]. Nevertheless, monomodular (16%), and trimodular (14%) alleles with long and short *C4B *gene variants are not uncommon [12].

Deletion or duplication of *CYP21A1P *or *CYP21A2 *together with *C4A *or *C4B *always occurs [8,13-15]. The deletion and large gene conversion were the first mutations described in 21-hydroxylase deficiency alleles [16-19]. Generally, disease-causing 30-kb deletions extend from 3'end *CYP21A1P *and include *C4B *and 5' end *CYP21A2 *to form inactive *CYP21A1P/A2 *chimeric genes [20-27]. Whereas monomodular alleles with *C4A/C4B *chimeric genes may also be formed after unequal crossovers, they are frequently associated to a functional *CYP21A2 *gene copy [13,22]. Such rearrangements can be recognized in Southern blots using *Taq *I restriction enzyme where *C4 *and *CYP21 *chimeric genes produce 6.4- and 3.2-kb fragments, respectively [22,23]. Deletions, large gene conversions and duplications may be evaluated either by Southern blot [3] or by the recently developed Multiplex Ligation-dependent Probe Amplification (*MLPA*) technique [24]. As *CYP21A1P/A2 *chimeric genes may carry different pseudogene-derived mutations, disease-causing deletion alleles may vary according the chimeric gene composition [21,22,25,26]. Novel *CYP21A1P/A2 *chimeric genes have been continuously described in different studies [27,28].

In Brazil, 30-kb deletion monomodular alleles correspond to ~9% of disease-causing alleles [29,30]. The present study describes the variability of such alleles in Brazilian patients (n = 20) with 21-hydroxylase deficiency using the conventional Southern blot, ASO-PCR and sequencing techniques combined to recently developed MLPA technique. Nine different and two novel monomodular haplotypes are reported. A 6.4-kb *Taq *I fragment corresponding to a *C4B/C4A *chimeric gene was unusually associated to *CYP21A1P/A2 *chimeric gene that also bore novel and rare single nucleotide polymorphisms (SNPs). Another allele was identified with a *CYP21A1P/A2 *gene carrying both p.P34L and p.H62L mutations in exon 1, however p.P30L, the most frequent pseudogene-derived mutation in this exon, was absent. The study of 59 healthy controls revealed that the novel p.P34L mutation is not pseudogene-derived and occurred only in alleles carrying the 30-kb deletion whereas the rare p.H62L mutation can be pseudogene-derived since it was found in 3.4% pseudogene sequences.

## Methods

### Subjects

This study was approved by the Ethics Committee from Universidade Estadual de Campinas (São Paulo, Brasil) and an informed consent was obtained from patients and relatives.

Twenty patients (8 males, 12 females) with 21-hydroxylase deficiency were included in this study (table 1). They represent 20 unrelated families. Consanguinity was reported in two families. Fourteen patients presented with the salt-wasting (SW) form, whereas five manifested the simple-virilizing (SV) form and one with the non-classic form (NC). Fifty-two family members were genotyped. Fifty non-related health individuals were screened for novel and rare mutations.

**Table 1:** Clinical data of CAH patients

Case	**Sex**^**1**^	**Age at diagnosis**^**2**^	Clinical Data	basal 17OHP (nmol/L)	**Na**^**+ **^**/K**^**+ **^**(mmol/L)**	**Phenotype**^**3**^
1	M	1 m 28 d	vomiting, dehydration	na^4^	129/6.2	SW
2	F	12 d	ambiguous genitalia; Prader III	> 20 ndm^5^	137/6.2	SW
3	M	2 m 24 d	vomiting, dehydration	49.3	116/9.2	SW
4	F	19 d	vomiting, dehydration; registered as male	na	128/7.8	SW
5	M	31 d	vomiting, dehydration	> 20 ndm	120/5.5	SW
6	M	2 m 8 d	vomiting, dehydration	> 200 ndm	121/6.8	SW
7	F	10 m	ambiguous genitalia; Prader III	> 25 ndm	142/5.0	SV
8	F	6 d	ambiguous genitalia; Prader III/IV	> 200 ndm	123/7.4	SW
9	F	np	ambiguous genitalia; Prader IV			SW
10	M	1 yr 1 m	vomiting, dehydration	> 25 ndm	120/6.8	SW
11	F	6 yr 3 m	precocious pubarche since 4 yr 6 m; high stature	> 25 ndm	nt	NC
12	F	10 d	ambiguous genitalia; Prader III	> 20 ndm	121/6.5	SW
13	F	np	ambiguous genitalia; Prader III/IV; vomiting, dehydration; registered as male	na	na	SW
14	F	np	ambiguous genitalia; Prader III	na	na	SW
15	F	14 d	ambiguous genitalia; Prader IV	> 6 ndm	119/5.3	SW
16	M	28 d	vomiting, dehydration, adrenal crysis	> 200 ndm	119/9.7	SW
17	M	3 yr 5 m	precocious pubarche since 2 yr	> 20 ndm	na	SV
18	M	6 yr	precocious pubarche	> 20 ndm	na	SV
19	F	5 yr 7 m	precocious pubarche, ambiguous genitalia; Prader IV	238	na	SV
20	F	37 d	ambiguous genitalia; Prader III	> 20 ndm	136/5.7	SV

^1^F = Female, M = male; ^2^yr = years, m = months, d = days, np = neonatal period; ^3^SW = salt-wasting, SV = simple virilizing, NC = non-classic; ^4^na = not available; ^5^ndm = non-diluted measurements (ref value 5.97 nmol/L).

### Methods

#### Southern blot and Allele-specific PCR (ASO-PCR)

**Figure 1:**
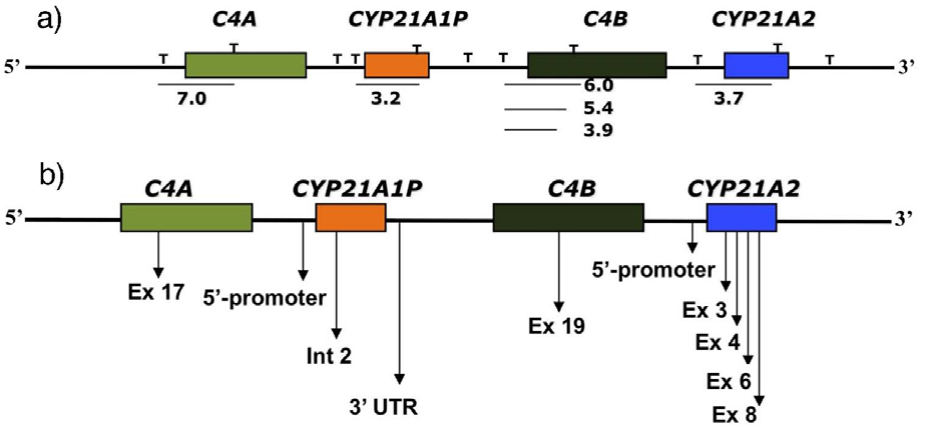
**Schematic representation illustrating the hybridization sites for *C4 *and *CYP21 *probes**. a) Southern blot: bars indicate hybridization regions for each probe; T denote *Taq *I restriction sites; numbers indicate sizes in kb of fragments recognized by each probe; b) MLPA experiment: arrows indicate the location where each probe hybridizes. Ex = exon, Int - intron.

Genomic DNA was obtained from peripheral blood by Proteinase K digestion and phenol/chloroform extraction [31]. Southern blots with *Taq *I digested DNA were performed following standard procedures [31]. Membranes were hybridized with *CYP21 *(pC21/3c) and *C4 *(C4B550) probes [29]. The probes were radiolabeled in a random priming reaction (Invitrogen, CA, USA) with α-^32^P-dCTP (Amersham, Uppsala, Sweden). The blots were washed using conventional conditions [31] and a final wash using 0.1 × SSC/0.1% SDS at 65°C was performed. Autoradiographies on Hyperfilm MP X-ray films (Amersham) were obtained. Blots were analysed following *Taq *I RFLP in *C4 *and *CYP21 *genes (Figure 1a) and relative band intensities.

ASO-PCR was performed with primers for the eight most common *CYP21A1P*-derived mutations as described elsewhere [32]. Six single nucleotide polymorphisms (SNPs) were also investigated either using ASO-PCR with primers specifically designed on basis the gene sequences or by digestion with restriction enzymes. Primers for exon 6 normal or mutant sequences (CL6N or Cl6M) were used as selective primers. As pseudogenes are also amplified in the analysis of *CYP21A1P/A2 *chimeric genes with exon 6 mutant primer, chimeric genes were identified upon analysis of all family members.

#### Multiplex Ligation-dependent Probe Amplification (MLPA)

MLPA was performed exactly as recommended by the manufacturers using SALSA MLPA P050B kit (MRC-Holland, Amsterdam, Netherlands) [33]. Fragment analysis was performed on an ABI 310 Genetic Analyzer (ABI PRISM/PE Biosystems, Foster City, CA, USA) and results were analyzed using Genescan and Genotyper softwares (Applied Biosystems, Foster City, CA, USA). Calculations were performed according to the method described by Taylor *et al. *[34]. The probemix included in the MLPA kit contains probes for several regions within *CYP21 *locus (Figure1b). Data were analysed using free Coffalyser MLPA data analysis software [33]. Normalized relative values ranging from 0.8 to 1.2 corresponded to two gene copies in the genotype, whereas values below 0.8 and above 1.2 corresponded to deletion (one gene copy) and duplication (three gene copies), respectively. This confidence interval was established by data obtained with five bimodular controls in each MLPA assay.

### Sequencing

A fragment including 600 bp from 5' promoter region to exon 6 (CL6N or CL6M) and a fragment from exon 6 to 3'UTR (CL6N or CL6M) were first amplified. The fragments were directly sequenced with internal primers using Big Dye™ Terminator Cycle Sequencing Kit V3.1 Ready Reaction (ABI PRISM/PE Biosystems). The sequences obtained in an *ABI 3700 *Automated Sequencer (ABI PRISM/PE Biosystems) were compared to *CYP21A2 *and *CYP21A1P *sequences (Ensembl - ENSG00000231852 and ENSG00000204338, respectively).

## Results

**Figure 2:**
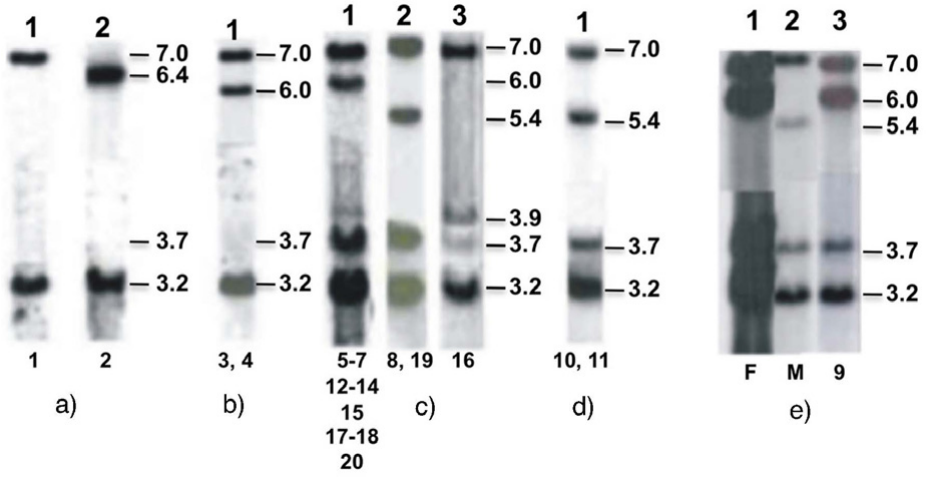
***Taq *I Southern blots showing patients carrying monomodular alleles**. Hybridizations with *CYP21 *(pC21/3c) and *C4 *(C4B550) probes are shown. a) Homozygous genotypes for monomodular alleles; b) compound heterozygous genotypes with mono- and bimodular alleles carrying large gene conversion; c) compound heterozygous genotypes with mono- and bimodular alleles carrying pseudogene-derived mutations; d) compound heterozygous genotype with mono- and trimodular alleles carrying pseudogene-derived mutations; e) compound heterozygous genotype with mono- and tetramodular alleles (lane 3); maternal genotype showing compound heterozygosis with mono- and bimodular alleles (lane 2); and paternal genotype with tetramodular and bimodular alleles (lane 1). Numbers below each lane depict patient numbers.

Southern blot analysis with *CYP21 *and *C4 *probes was performed to distinguish deletions from large gene conversions. ASO-PCRs using CL6N or CL6M sequences as selective primers were performed to investigate both pseudogene-derived mutation and polymorphisms present in chimeric *CYP21A1*/*A2 *and *CYP21A2 *genes. All family members have been analyzed; therefore, pseudogenes and chimeric genes carrying CL6M sequences could be distinguished. Southern blot revealed 30-kb deletion in 20 patients with different genotypes (table 2; Figure 2).

**Table 2:** Genotypes of CAH patients

Patients	Genotype	
	
	Paternal Allele	Maternal Allele
1	30-kb del^1^	30-kb del
2	30-kb del	30-kb del
3	LGC2	30-kb del
4	30-kb del	LGC
5	IVS2-2A > G	30-kb del
6	30-kb del	c.920_921insT
7	p.I172N	30-kb del
8	30-kb del	p.P30L
9	p.W19X	30-kb del
10	p.Q318X	30-kb del
11	30-kb del	p.V281L
12	30-kb del	IVS2-13A/C > G
13	p.R356W	30-kb del
14	IVS2-13A/C > G	30-kb del
15	c.920_921insT	30-kb del
16	30-kb del	p.R356W
17	p.I172N	30-kb del
18	30-kb del	p.Q318X
19	30-kb del	p.I172N
20	30-kb del	IVS2-13A/C > G

^1^30-kb del = deletion of 30 kb including 3'-end *CYP21A1P, C4B *and 5'-end *CYP21A2*,

^2^LGC = large gene conversion;

**Figure 3:**
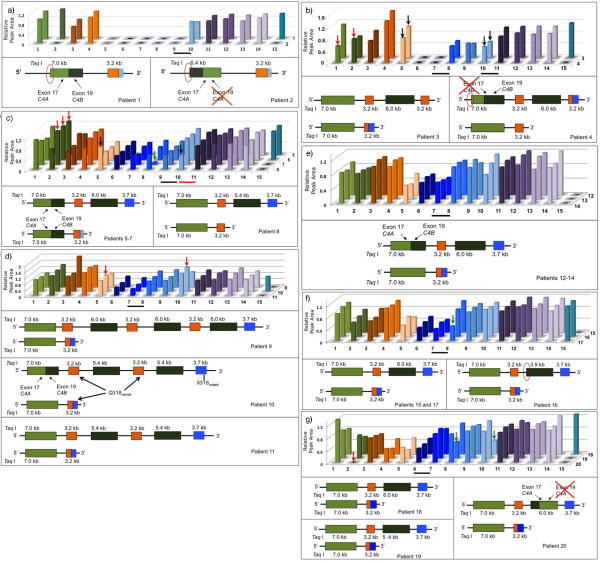
**Integrated and normalized MLPA data**. *C4A*, *C4B, CYP21A1P*, *CYP21A2*, *TNXB*, *CREB1 *are shown. Numbers 1-15 correspond to: (1) *C4A *exon 17, (2) *C4B *exon 19, (3-5) *CYP21A1P *5' promoter region, intron 2, 3'UTR; (6-10) *CYP21A2 *5' promoter region, exon 3, exon 4, exon 6, exon 8; (11-13) *TNXB *exon 32, 15, 1; (14) *CREB1 *probe. (15) Y-chromosome probe. Horizontal black bars denote recombination breakpoints (RB); red bar, for patient 8. Columns correspond to integrated and normalized electropherogram peak areas, values between 0.8 and 1.2 indicate two copies, below and above correspond to one or more than two copies, respectively. Patients' numbers are on the right. Green arrows indicate heterozygosis for p.I172N and p.Q318X (patient 18). Black arrows indicate 2:1 ratio for two copies of 3'UTR pseudogene (5) and one copy of exon 8 (10). (a-g) Upper panels - MLPA results; lower panels - schematic genotypes. a) Monomodular homozygosis; red circles denote different *C4 *5'-end; b) Mono- and bimodular alleles carrying chimeric genes; red arrows indicate 1:2 ratio (*C4A:C4B*); c) heterozygosis for mono- and bimodular alleles; red arrows indicate three copies of *C4B*; d) heterozygosis for mono- and tri- or tetramodular alleles; red arrows indicate 2:2 ratio for 3'-end *CYP21A1P *(5) to exon 8 *CYP21A2 *(10); patient 10 - trimodular allele bearing normal p.Q318 in the two pseudogenes; e) heterozygosis for alleles mono- and bimodular with *CYP21A1P/A2 *and *C4A/C4B*, respectively; f-g) heterozygosis for mono- and bimodular alleles; RB between exons 3-4 and 1-3, respectively; red arrow - null *C4B *hybridization signal (patient 20).

MLPA technique was used to confirm Southern blot results and to obtain data on the composition of chimeric genes since it includes one probe for each *C4A *and *C4B *genes, and three and five probes, respectively, for *CYP21A1P *and *CYP21A2 *genes (Figure 1b). The MLPA kit available also analyzes *TNXB *located within the RCCX locus and *CREBL1 *on chromosome 6p21.3 and nineteen control genes as well. For all patients, normalized data showed two copies for *TNXB *and *CREBL1 *genes (Figure 3) on chromosome 6 and also for control genes (data not shown) indicating no copy number variation in other loci.

After sequencing *CYP21A1P*/*A2 *chimeric genes, novel mutations and novel single nucleotide polymorphisms (SNPs) have been identified. Haplotypes were classified according to *C4 *gene characteristics and SNPs and mutations present in *CYP21A1P/A2 *chimeric genes. Table 3 shows nine different haplotypes for monomodular alleles identified in this study.

**Table 3:** Haplotypes for monomodular alleles defined on basis of C4 and CYP21A1P/A2 chimeric gene

Haplotypes			I	II				III	IV	V				VI			VII			VIII		IX
**Patients**			**2**	**1**	**5**	**6**	**7**	**4**	**8**	**3**	**10**	**15**	**16**	**9**	**11**	**12**	**13**	**14**	**17**	**18**	**19**	**20**

C4		Sb^1^	6.4	7	7	7	7	7	7	7	7	7	7	7	7	7	7	7	7	7	7	7
		MLPA^2^	B/A	A/B	A/B	A/B	A/B	A	A	A	A	A	A	A	A	A	A	A	A	A	A	A

CYP21A1P/A2	5'	RB^3^	6/8	6/8	6/8	6/8	6/8	3'end	3'end	3/4	3/4	3/4	3/4	3/4	3/4	3/4	3/4	3/4	3/4	1/3	1/3	1/3
		-449^4^	A	A	A	A	A	A	-	A	A	A	A	A	A	-	G	G	G	A	A	A
		-308^5^	C	C	C	C	C	C	-	G	G	G	G	C	C	-	C	C	C	C	C	C
		-289^6^	C	T	T	T	T	T	-	T	T	T	T	T	T	-	T	T	T	T	T	T
		-4^7^	T	C	C	C	C	T	T	C	C	C	C	T	T	T	T	T	T	T	T	T
	
	E1	p.P30L^8^	T	T	T	T	T	T	T	C	C	C	C	-	T	T	T	-	T	T	T	T
		p.P34L^9^	C	C	C	C	C	C	C	T	T	T	T	-	C	C	C	-	C	C	C	C
		p.H62L^10^	A	A	A	A	A	A	A	T	T	T	T	-	A	A	A	A	A	A	A	A
	
	I2	395^11^	T	C	C	C	C	T	T	C	C	C	C	C	C	C	T	T	T	C	C	C
		419^12^	-	-	-	-	-	C	C	C	C	C	C	C	C	C	C	C	C	C	C	A
		547^13^	-	-	A	-	-	A	A	A	A	-	A	A	A	A	A	-	A	A	A	C
		560_566insG^14^	-	6Gs	6Gs	-	6Gs	6Gs	6Gs	6Gs	6Gs	-	6Gs	6Gs	6Gs	6Gs	6Gs	-	6Gs	6Gs	6Gs	7Gs
		602^15^	A	A	A	A	A	A	A	A	A	-	A	A	A	A	A	-	A	C	C	C
	
	E4	p.I172N	A	A	A	A	A	A	A	T	T	T	T	T	T	T	T	T	T	T	T	T
	
	I5	1253^16^	A	G	G	G	G	G	G	G	G	G	G	G	G	G	G	G	G	-	-	-
	
	E7	p.S268T^17^	G	C	C	C	C	C	G	G	G	G	G	G	G	G	G	G	G	G	G	G
		p.V281L^18^	T	T	T	T	T	T	T	G	G	G	G	G	G	G	G	G	G	G	G	G
	
	E8	p.Q318X^19^	C	C	C	C	C	T	T	C	C	C	C	C	C	C	C	C	C	C	C	C
		p.R356W^20^	C	C	C	C	C	C	T	C	C	C	C	C	C	C	C	C	C	C	C	C

Numbers are relative to ATG initiation codon in the genomic DNA based on CYP21A2 sequence described by Higashi et al. (1986); mutation caused by nucleotide changes; E, exon; I, intron. ^1^Sb, size (kb) of *C4 *Taq I restriction fragments obtained in Southern blots. ^2^Composition of the *C4 *gene copy in monomodular alleles indicated by MLPA assays - A, corresponds to *C4A *and B, to *C4B *genes. ^3^RB recombination breakpoint in *CYP21A1P/A2 *chimeric gene indicated by MLPA and ASO-PCR experiments; 6/8, between exons 6 an 8; 3/4, between exons 3 an 4; 1/3, between exons 1 an 3. *CYP21A1P */*CYP21A2 *consensus nucleotides for polymorphic positions (number at NCBI-SNP database), most frequent nucleotide for each SNP is indicated first: ^4^A > G (rs28361032)/A (non-polymorphic); ^5^C (non-polymorphic)/C > G (rs3130676); ^6^T (non-polymorphic)/T (non-polymorphic); ^7^T > C (rs6470)/C > T (rs6470); ^8^T > C (rs9378251)/C > T (rs9378251); ^9^C (non-polymorphic)/C (non-polymorphic); ^10^A > T (rs9378252)/A > T (rs9378252); ^11^C > T (rs28361033)/T > C > G (rs6462); ^12^A (non-polymorphic)/A > C (rs6448); ^13^A(non-polymorphic)/C (non-polymorphic); ^14^6Gs (non-polymorphic)/7Gs > 6Gs (not registered); ^15^A(non-polymorphic)/C > A > G (rs6451); ^16^G > A (rs28691121)/G (non-polymorphic); ^17^G (non-polymorphic)/G > C (rs6472); ^18^T > G (rs41315836)/G > T (rs6471); ^19^T > C (rs7755898)/C > T (rs7755898); ^20^T > C (rs7755898)/C > T (rs7769409).

Patients 1 and 2, who were children of consanguineous marriages, were homozygous for a monomodular allele. However, hybridization results with C4 probe indicated a 6.4-kb *Taq *I fragment in patient 2, whereas patient 1 presented the usual 7.0-kb *C4A *fragment (Figure 2a). Molecular investigation on patient's 2 family demonstrated that the novel allele has been transmitted through three generations (data not shown). MLPA indicated similar composition of *CYP21A1P*/*A2 *chimeric genes in both cases (Figure 3a, upper panel), but once again the two patients differed in the *C4 *gene composition (Figure 3a, lower panels). Patient 2, who carried the 6.4-kb *Taq *I fragment, presented MLPA signal for two copies only for *C4A *probe, whereas patient 1, who carried the 7.0-kb *C4A Taq *I fragment, showed signals for two copies of *C4A *and *C4B *probes, suggesting that both *C4 *in the genotype have exon 19 sequences corresponding to *C4B *gene. Therefore, the two different *C4 *sequence compositions indicate distinct 30-kb deletion alleles. Patient 1 showed haplotype II (table 3) that, besides the C4A/B chimeric copy, also presented the C allelic variant at both -4 (SNP-rs6470) and g.395 (SNP-rs6462) nucleotides. Haplotype I is unique for patient 2 (table 3). In addition to the C4B/A^[6.4] ^gene, it carries the novel -289T > C SNP in *CYP21A1P*/*A2 *promoter region. The T variant at g.395 (SNP-rs6462) nucleotide and the rare pseudogene-derived g.1253A variant (SNP-rs28691121) were also detected (table 3).

Patients 3 and 4 presented Southern blot results compatible with compound heterozygosis for 30-kb deletion and large gene conversion in which no signal for 3.7-kb *CYP21A2 *fragment was observed, whereas *C4A*^[7.0] ^and *C4B*^[6.0] ^fragments were present with intensities of 2:1 (Figure 2b). In MLPA experiments *C4A:C4B *ratios for patients 3 and 4 were 2:1 and 1:2, respectively (Figure 3b, upper panel). Both showed MLPA profiles compatible with mono-/bimodular compound heterozygosis with two different *CYP21A1P*/*A2 *chimeric genes (Figure 3b). However, the composition of monomodular allele in each case could only be defined after MLPA analysis of their parents (data not shown). For patient 3, the breakpoint between exon 3 and 4 in the chimeric *CYP21A1P*/*A2 *gene was defined for the paternal inherited monomodular allele. The maternal inherited bimodular allele carrying large gene conversion presented a complete pseudogene sequence indicated by the 2:1 ratio for probes *CYP21A1P*-3'end and *CYP21A2*-exon 8 in MLPA (Figure 3b, left lower panel) instead of the 1:2 expected if it was a chimeric gene with *CYP21A2-*3'end. Similar results were obtained for patient 4 except that in this case the complete pseudogene was concluded to be associated to the monomodular allele, whereas the chimeric *CYP21A1P*/*A2 *gene with recombination breakpoint between exon 3 and 4 was associated to the bimodular allele with large gene conversion (Figure 3b, right lower panel). In addition, based on MLPA analysis of her parents (data not shown), the maternal bimodular allele in patient 4 demonstrated to carry one of *C4 *copies in which *C4A *probe could not hybridize; therefore the 1:3 ratio observed in her mother indicated absence of *C4A *exon 17 sequences and presence of *C4B *exon 19 sequences which is suggestive of a chimeric *C4A/B *gene or a gene conversion at exon 17 (Figure 3b, right lower panel). Considering *C4 *composition and *CYP21A1P/A2 *SNPs, the 30-kb deleted alleles in patients 3 and 4 correspond to haplotypes V and III, respectively (table 3). Haplotype V is defined by the presence of the novel p.P34L and the rare p.H62L mutations, but absence of the pseudogene-derived p.P30L, in exon 1. The variant -308G and the variant C at -4 and g.395 nucleotides characterized this haplotype in which the recombination breakpoint was located between exon 3 and 4.

For other sixteen patients (5-20) who were compound heterozygous with monomodular and bi-, tri- or a probable tetramodular alleles bearing pseudogene-derived mutations in the *CYP21A2 *gene (Figure 2c-e; table 2), different *C4A *to *C4B *and *CYP21A1P *to *CYP21A2 *probe ratios in MLPA were observed (Figure 3c-g). These patients were divided in two major groups according to the status of exon 6 in the *CYP21A1P*/*A2 *chimeric genes: patients 5-8 with mutant exon 6 are shown in Figure 3c, and patients 9-20 with normal exon 6 in Figure 3d-g.

Chimeric *CYP21A1P*/*A2 *gene composition for alleles in patients 5-7 seemed to be similar to that in patients 1 and 2 with mutant exon 6 and normal exon 8 (Figure 3a-c, upper panels). However, *C4A*:*C4B *ratios were 2:3, suggesting that both *C4A *copies may carry exon 19 corresponding to *C4B *gene similar to that observed for patient 1. MLPA results for each family (data not shown) indicated the segregation of both *C4 *and *CYP21 *chimeric genes in the same allele (Figure 3c, left lower panel). Patient 8 presented 2:1 MLPA ratio for *C4A:C4B *sequences in agreement with Southern blotting. MLPA showed that 21-hydroxylase gene copy in this case corresponded to a complete pseudogene as the ratio for probes *CYP21A1P *3'-end and *CYP21A2-*exon 8 was 2:1 as described above for patients 3 and 4 (Figure 3c, right lower panel). SNPs in chimeric genes classified the monomodular alleles in patients 5-7 within haplotype II (table 3). Besides deletion, duplication and chimeric gene composition, MLPA analyzes could identify compound heterozygosis for p.I172N in patient 7 (table 2; Figure 3c), because the probe used for *CYP21A2 *exon 4 anneals to the normal sequence at codon 172. Patient 8 showed haplotype IV, which can be differentiated from III at the SNPs g.1645G > C (SNP-rs6472; p.S268T) and at the g.2110C>T (p.R356W) mutation (table 3).

MLPA results for patients 9-20 were shown in Figure 3d-g. Additionally, the probable localization of recombination breakpoint within *CYP21A1P*/*A2 *chimeric genes divided them in two groups: patients 9-17 with breakpoint between exon 3 and 4 (Figure 3d-f) and patients 18-20 between exon 1 and intron 2 (Figure 3g). A combination of mono- and a probable tetramodular allele was considered for patient 9 after analysis of allelic segregation in the family (Figure 2e). This was the most probable combination to explain the relative intensity for *C4A*^[7.0]^:*C4B*^[6.0] ^(2:3) and for *CYP21A2*^[3.7]^:*CYP21A1P*^[3.2] ^(1:4) fragments in the patient. The mother presented ratios of 2:1 for *C4A*^[7.0]^:*C4B*^[5.4] ^and 1:3 for *CYP21A2*^[3.7]^:*CYP21A1P*^[3.2] ^and was positive for p.P30L, IVS2-13A/C > G and Δ8 mutations in ASO-PCR experiments indicating a deleted allele with CL6N sequence. The daughter inherited the 30-kb deleted allele from the mother, therefore she should carry an allele with three copies of each *C4B *and *CYP21A1P *to produce the relative intensities observed in the Southern blot (Figure 2e, lanes 2 and 3). Patients 10 and 11 were compound heterozygotes for tri- and monomodular alleles. These genotypes may explain the 2:2 ratio for *C4A *and *C4B *genes observed in Southern blot (Figure 2d). However, a 2:2 ratio for *C4A:C4B *in MLPA experiments has been observed only for patient 11. Patient 10 showed a 2:3 gene ratio, which is probably due to a positive hybridization signal for exon 19 *C4B *probe within the *C4A *gene copy in the paternal trimodular allele, deduced from the family analysis, therefore his most probable genotype is shown in Figure 3d (middle panel). An additional difficulty in interpreting MLPA results for patient 10 was that he also carried p.Q318X in *CYP21A2 *gene copy of the paternal inherited trimodular allele indicated in ASO-PCR experiments (table 2). As *CYP21A2 *exon 8 MLPA probe anneals to the normal sequence at codon 318, the result expected in this case was a ratio of 2:1 for probes *CYP21A1P *3'UTR to *CYP21A2 *exon 8, if both pseudogenes in the trimodular allele were mutant in codon 318. However a ratio 2:3 was obtained. Analysis of his parents (data not shown) indicated that the two pseudogenes in the paternal trimodular allele might carry the normal sequence in codon 318 (Figure 3d, middle panel). The monomodular alleles carried by patients 10 and 11 were classified, respectively, within haplotype V and VI (table 3).

Patients 12-14 presented 2:2 MLPA ratios for *C4A:C4B *contrasting with the 2:1 obtained on Southern blot (Figure 2c). In these cases, familial analysis demonstrated that the bimodular alleles they carry in compound heterozygosis with monomodular alleles, probably, contain *C4A *genes formed by 5'-end *C4A*^[7.0]^, *C4A *exon 17, but exon 19 corresponds to *C4B *specific sequences (Figure 3e). The monomodular allele in patient 12 corresponds to haplotype VI, whereas patients 13 and 14 carry haplotype VII (table 3). Haplotype VI differs from VII by the -449A>G (SNP-rs28361032) and g.395T > C (SNP-rs6462).

Patients 15-19 demonstrated *C4A*:*C4B *ratios of 2:1 in both Southern blotting and MLPA (Figure 3f-g). A 3:0 ratio in MLPA for *C4A:C4B *was observed for patient 20, whereas Southern blotting showed a ratio of 2:1. This result might indicate a *C4A *gene copy with a 6.0-kb Taq I fragment in which exon 17 sequences correspond to *C4A *and *C4B *specific sequences at exon 19 are absent, therefore the most probable genotype in this case is shown in Figure 3g (right lower panel). MLPA indicated compound heterozygosis for p.I172N in patients 17 and 19 (table 2; Figure 3f,g). Likewise, it also detected compound heterozygosis with p.Q318X mutation in patient 18 (table 2; Figure 3g). The recombination breakpoint indicated in MLPA for patients 15-17 was between exon 3 - 4 (Figure 3f). Sequencing data indicated haplotype V for alleles in patients 15 and 16 and haplotype VII for patient 17 (table 3). The recombination breakpoint indicated in MLPA for patients 18-20 was between exon 1 and intron 2 (Figure 3f-g). However, sequencing results suggested distinct alleles within this group. In haplotype VIII, which is shared by patients 18 and 19, the recombination breakpoint was probably located between the SNP 560_566insG and 602A > C (SNP-rs6451) as shown in table 3. Whereas, the recombination breakpoint for the allele carried by patient 20 (table 3, haplotype IX) was probably located at the beginning of intron 2 since it carried the variant A at the g.419 position (SNP - rs6448), which is more frequent in *CYP21A2 *gene and the nucleotide C at 547 position that is characteristic of the *CYP21A2 *gene.

Sequencing 170 *CYP21A2 *genes from obligate heterozygote individuals and *CYP21A1P *sequences from 59 control individuals indicated that p.P34L was not present in any gene or pseudogene sequences, whereas heterozygosis for p.H62L was observed in the pseudogene of four individuals (data not shown). Therefore, p.P34L seems to be a novel mutation not derived from pseudogene, whereas p.H62L showed a frequency of 3.4% in pseudogenes.

## Discussion

This study reports two novel deletion alleles in Brazilian patients with 21-hydroxylase deficiency and describes the variability of *C4/CYP21 *monomodular alleles evaluated by combining Southern blot, ASO-PCR, MLPA and sequencing techniques. The occurrence of different chimeric genes in Brazilian patients had not been reported before. Although Real-Time PCR and HLA-haplotypes would be alternative techniques to estimate, respectively, *C4 *and *CYP21A2 *copy number [35-37] and founder effect they were not available for this work.

By combining different techniques we characterized monomodular alleles carrying CYP21A1P/A2 chimeric genes in genotypes that were compound heterozygous with bi - or trimodular alleles bearing pseudogene-derived mutations, including a bimodular allele carrying p.R356W mutation associated to the rare 3.9-kb *C4B *fragment (table 2, patient 16; Figure 2c), described before in a non-disease causing allele [38]. Patient 9 who inherited from the mother the monomodular allele might also carry a tetramodular allele inherited from the father, however a more detailed study is required to confirm the tetramodular configuration for this allele that also carry a novel g.60G > A (TGG > TGA) nucleotide change causing the p.W19X mutation. The same mutation had been described before as a result of the TGG > TAG nucleotide change [39].

In general, data were convergent independently on the technique used. However, patients with alleles carrying a *C4B/A *or *C4A/B *gene and those in compound heterozygosis with trimodular alleles could be misinterpreted as carrying large gene conversions if only MLPA had been used, because C4 probes resulted in signals indicative of alleles without *C4B *deletion. Patients 1 and 2, are typical examples. The 6.4-kb *Taq *I fragment identified in patient 2 is generally a marker for a deletion that results from unequal crossover between a *C4A*^[7.0] ^and a short *C4B*^[5.4] ^gene [26,40]. Such fragment is indicative of *CYP21A1P *deletion in monomodular alleles. It is found with a frequency of 11% in the general population and do not cause 21-hydroxylase deficiency [12] and would not be recognized in MLPA assay. The 6.4-kb gene variant is here described for the first time in association with a *CYP21A1P/A2 *gene in a case of 21-hydroxylase deficiency. Alleles bearing *CYP21A1P *deletions have been proposed as premutation for generating *CYP21A2 *deleted alleles [41]. It can be speculated that an allele bearing *C4B/A*^[6.4] ^and *CYP21A1P *deletion might have influenced the formation of the monomodular allele in which the deleted allele seems to have been generated in at least two steps of unequal crossovers. However, those events must have happened several generations ago because the mutated allele had been segregating in this family for three generations. Additionally, those recombination events might have introduced novel nucleotide variants such as the -289T > C SNP in the 5'-promoter region of *CYP21A1P*/*A2*. The T variant at nucleotide 395, which is most frequent in *CYP21A2 *gene and a rare 1253G > A SNP within intron 5, which is described only in the *CYP21A1P *as a rare polymorphic nucleotide position, were also identified in this *CYP21A1P/A2 *chimeric gene. Therefore haplotype I was uniquely found in patient 2. In a paper published before [42], data obtained with ELISA assays also showed differences in C4 protein levels between patients 1 and 2. Low level of C4A protein was observed for patient 1 whereas patient 2 showed low level of C4B protein. Although *C4A *and *C4B *genes share 99% sequence identities, the proteins they encode have different hemolytic activities, covalent affinities to antigens and immune complexes, and serological reactivities [12,43]. *C4A *gene copies producing proteins with electrophoretical characteristics of C4A but acting antigenically as C4B have been described in association to 30-kb deletion haplotypes [20,44]. Differences conferring specific binding affinities are located within exon 26 [45], therefore monomodular allele in patient 1 may have 5'-end of *C4A *as indicated by 7.0-kb fragment and *C4B *sequences from exon 19 to 3'-end as indicated by MLPA and ELISA assays. Similarly, patients 5, 6 and 7 also carried a *C4A*^[7.0] ^fragment with 3'-end *C4B *sequences as denoted by Southern blot and MLPA results. These patients together with patient 1 grouped within haplotype II, which is similar to those described in several populations [15,20,46,47]. In addition, patient 20 showed positive signal for *C4A *gene but no signal for *C4B *probe (3:0) in MLPA. This result indicate that exon 17 in the *C4B*^[6.0] ^gene probably corresponded to *C4A *sequences and the patient had been described as having very low immune response toward C4B antiserum [42]. Although this allele was not associated to 30-kb deletion, it illustrates C4 gene variability that influences MLPA results.

Both haplotypes III and IV carry a complete *CYP21A1P *pseudogene copy indicating that the recombination breakpoint should map within the 5 kb that separate 3'-end *CYP21A2 *and the exon 32 of *TNXB *gene. They differed because haplotype III showed the C allelic variants in both g.1645 and g.2110 (p.R356W) nucleotides whereas G and T, respectively, were observed in haplotype IV (table 3). Polymorphisms in *CYP21A1P *pseudogene are known to be common [40], therefore those haplotypes could be originated from unequal crossovers involving bimodular alleles carrying different pseudogene variants. Recombination within 3'-end *CYP21A2 *seems to be frequent since it has been reported by different research groups [15,41] including as a *de novo *event in which deleted alleles were generated [48,49].

A group of ten genes had recombination breakpoints identified between exon 3 and 4. Haplotype V diverged from the VI and VII because the alleles did not carry the pseudogene-derived p.P30L, but showed p.P34L and p.H62L mutations in exon 1. p.H62L has been recently described in association with p.P453S in patients with the NC form of 21-hydroxylase deficiency [50,51]. In order to investigate whether p.P34L and p.H62L were rare pseudogene-derived mutations that have arisen in this chimeric gene through unequal crossover between gene and pseudogene, since they are flanked by *CYP21A1P*-like sequences, we tested 170 *CYP21A2 *genes from obligate heterozygous individuals and *CYP21A1P *sequences from 59 control individuals. Here we demonstrated that p.H62L might be originated in *CYP21A1P *since it was found in 3.4% pseudogene sequences. However, p.P34L was not found in any pseudogene sequences, therefore its origin remains unclear. Also, this haplotype showed the G variant at nucleotide -308 in the *CYP21A1P/A2 *5'-promoter region. Interestingly, this position is considered polymorphic only in *CYP21A1P *with frequencies varying from 0.1% to 0.3% in Chineses and Africans from Nigeria (NCBI-SNP database). Different mutations in exon 1 together with rare 5'-end SNP characterize a novel haplotype that seems to be frequent in Brazilian patients carrying monomodular alleles since it corresponded to 20% (4/20) of total 30-kb deleted alleles reported here as causing 21-hydroxylase deficiency. Probably, haplotype V have arisen in Brazil from an African ancestral since Brazilian population is highly miscigenated with populations from different origins including Africans of Benin Gulf region [52]. This haplotype might have segregated with 21-hydroxylase deficiency through an isolated founder effect for 30-kb deletion alleles. Main differences between haplotype VI and VII was found in -449 and g.395 nucleotides (table 3). The -449 position is considered to be ambiguous only in the pseudogene (SNP # rs28361032) whereas 395 is polymorphic in both gene and pseudogene sequences (SNP # rs6462) but there are no frequency data for the any of their allelic variants.

Haplotypes VIII and IX were separated because they showed different recombination breakpoints within intron 2. Those two haplotypes presented the *CYP21A1P *promoter region and p.P30L mutation as pseudogene-derived sequences and they are not very frequent in other populations [15,53].

CYP21A1P/A2 chimeric genes can be correlated with SW or SV forms of 21-hydroxylase deficiency depending on the mutations they carry [15,20,46,47]. The variability verified among 30-kb alleles with CYP21A1P/A2 chimeric genes present in different genotypes did not influence the phenotypes observed (tables 1, 2). Therefore, patients 1-6, 9-10 and 12-16 presented SW form of 21-hydroxylase deficiency as expected for homozygosis or compound heterozygosis for haplotypes I-VII and mutations that severely affect the enzyme activity (tables 1, 2). Similarly, compound heterozygote patients for haplotypes I-VII and p.I172N or p.V281L mutations presented, respectively, SV or NC forms of the disease (tables 1, 2, patients 7, 11, 17). Patients carrying haplotypes VIII and IX presented SV phenotype independently if they were compound heterozygous with a SV (tables 1, 2; p.I172N, patient 19) or SW mutations (tables 1, 2; p.Q318X and IVS2-13A/C > G, patients 18 and 20, respectively) indicating a synergistic effect of *CYP21A1P *promoter region and p.P30L upon the enzyme activity as proposed before [54].

Our study showed that the combination of Southern blot and ASO-PCR/direct sequencing with MLPA tests may constitute an option for mapping and better characterize chimeric genes on RCCX monomodular alleles, especially in populations with high allelic diversity such as that in Brazil. MLPA has been proposed as a candidate with good potential to be used in neo-natal screening and in pre-natal diagnosis because it can be performed with very low amount of DNA [55]. Eventually, MLPA may substitute time-consuming Southern blot in cases were HCA diagnosis is urgent as it managed to estimate the *CYP21A1P/A2 *borders in almost all cases. However, to distinguish between deletions and large gene conversions in genetic studies searching for detailed allelic information, it would be more informative if more *C4 *and *CYP21 *probes were included in the analysis.

## Conclusions

Our study showed that the combination of Southern blot and ASO-PCR/direct sequencing with MLPA tests may constitute an option for mapping and better characterize chimeric genes on RCCX monomodular alleles, especially in populations with high allelic diversity such as that in Brazil. MLPA has been proposed as a candidate with good potential to be used in neo-natal screening and in pre-natal diagnosis because it can be performed with very low amount of DNA [55]. Eventually, MLPA may substitute time-consuming Southern blot in cases were HCA diagnosis is urgent as it managed to estimate the *CYP21A1P/A2 *borders in almost all cases. However, to distinguish between deletions and large gene conversions in genetic studies searching for detailed allelic information, it would be more informative if more *C4 *and *CYP21 *probes were included in the analysis.

## Competing interests

The authors declare that they have no competing interests.

## Authors' contributions

FBC carried out part of Southern blot, the MLPA and sequencing experiments and sequence alignment; FCS, RDB and RJP participated in ASO-PCR experiments for pseudogene-derived mutations and SNPs; MA, LCP and IFL participated in Southern blot and AS0-PCR studies of seven families; SHVLM, MTMB and GGJ were responsible for diagnosis and management of patients and participated in the design of the study; MPM conceived the study, and participated in its design and coordination and also drafted the manuscript. All authors read and approved the final manuscript.

## Pre-publication history

The pre-publication history for this paper can be accessed here:

http://www.biomedcentral.com/1471-2350/11/104/prepub
